# Photosensitivity of Different Nanodiamond–PMO Nanoparticles in Two-Photon-Excited Photodynamic Therapy

**DOI:** 10.3390/life12122044

**Published:** 2022-12-07

**Authors:** Nicolas Bondon, Denis Durand, Kamel Hadj-Kaddour, Lamiaa M. A. Ali, Rabah Boukherroub, Nadir Bettache, Magali Gary-Bobo, Laurence Raehm, Jean-Olivier Durand, Christophe Nguyen, Clarence Charnay

**Affiliations:** 1 ICGM, University of Montpellier, UMR-CNRS 5253, 34293 Montpellier, France; 2 IBMM, University of Montpellier, UMR-CNRS 5247, 34293 Montpellier, France; 3 Univ. Lille, CNRS, Centrale Lille, Univ. Polytechnique Hauts-de-France, UMR 8520-IEMN, 59000 Lille, France

**Keywords:** nanodiamond, two-photon excitation, mesoporous organosilica, theranostics, cancer cells

## Abstract

Background: In addition to their great optical properties, nanodiamonds (NDs) have recently proved useful for two-photon-excited photodynamic therapy (TPE-PDT) applications. Indeed, they are able to produce reactive oxygen species (ROS) directly upon two-photon excitation but not with one-photon excitation; Methods: Fluorescent NDs (FNDs) with a 100 nm diameter and detonation NDs (DNDs) of 30 nm were compared. In order to use the gems for cancer-cell theranostics, they were encapsulated in a bis(triethoxysilyl)ethylene-based (ENE) periodic mesoporous organosilica (PMO) shell, and the surface of the formed nanoparticles (NPs) was modified by the direct grafting of polyethylene glycol (PEG) and amino groups using PEG-hexyltriethoxysilane and aminoundecyltriethoxysilane during the sol–gel process. The NPs’ phototoxicity and interaction with MDA-MB-231 breast cancer cells were evaluated afterwards; Results: Transmission electronic microscopy images showed the formation of core–shell NPs. Infrared spectra and zeta-potential measurements confirmed the grafting of PEG and NH_2_ groups. The encapsulation of the NDs allowed for the imaging of cancer cells with NDs and for the performance of TPE-PDT of MDA-MB-231 cancer cells with significant mortality. Conclusions: Multifunctional ND@PMO core–shell nanosystems were successfully prepared. The NPs demonstrated high biocompatibility and TPE-PDT efficiency in vitro in the cancer cell model. Such systems hold good potential for two-photon-excited PDT applications.

## 1. Introduction

Nanodiamonds (NDs) with tunable surface chemistry and core defect composition can be prepared via a large range of processes [1,2]. The luminescence properties of NDs are generally correlated with the proportion of nitrogen vacancy (NV) centers in the nanocrystals. NDs are thus extensively reported as bioimaging probes. However, their application as photoactive species for therapy has been described in only a few recent studies [3,4,5,6,7,8]. As with other carbon-based semiconductors (e.g., carbon quantum dots or single-walled carbon nanotubes), NDs are mostly known as electron-donor agents for photodynamic and photothermal therapies, combined with phthalocyanine or porphyrin photosensitizers, but not as direct sensitizing drugs.

NDs’ ability to generate reactive oxygen species (ROS) for two-photon-excited photodynamic therapy (TPE-PDT) was demonstrated recently for the first time. Briefly, both NDs alone and NDs covered by an ethenylene-based periodic mesoporous organosilica (PMO) shell were irradiated at 800 nm with a pulsed femtosecond laser and induced 32 ± 6 and 39 ± 10% mortality on MCF-7 breast cancer cells, respectively [5]. Moreover, PMO nanoparticles (NPs) without NDs displayed no toxicity upon two-photon irradiation, which is characteristic of organosilica-based materials [9,10]. Negatively charged nitrogen vacancy (NV^−^) centers, located in the cores of the diamond particles, are probably the origin of the TPE-PDT effect observed. As expressed by the energy-level diagram of such centers (Figure S1), the zero-phonon line (ZPL) in NV^−^ centers between a ground state ^3^A_2_ and an excited state ^3^E equals 1.95 eV (637 nm) [11]. When performing two-photon irradiation at 800 nm (1.55 eV/photon), the selection rules allow the electronic transition from a ground to an excited state, with the absorption energy calculated as 2.33 eV (533 nm) [12]. Then, the TPE-PDT principle is applicable to nonradiative degeneration from the m = ± 1 sublevels of ^3^E to ^1^E’ by intersystem crossing (ISC) [13]. Finally, the transition from ^1^E’ to ^1^A_1_ could result in ROS production through a reaction with the surrounding oxygen in biological media. Furthermore, the encapsulation of different nanoparticles (gold nanoparticles [14], nanoprisms [15], and nanodiamonds [5]) in mesoporous organosilica has been performed in order to enhance the biocompatibility of the nanosystems.

In this context, the TPE sensitivities of two different commercially available hydroxylated NDs were assessed after their encapsulation in an ethenylene-based (ENE) PMO shell that can enhance the transfer of electron/hole pairs. The selected NDs exhibit variable NV^−^ centers contents, sizes, and shapes, which can influence their optical properties [16]. The nanomaterials’ preparation and characterization, as well as their in vitro killing efficiency upon TPE-PDT will be presented in the next sections. To the best of our knowledge, this early study is the first report comparing different encapsulated NDs displaying two-photon properties for photodynamic therapy.

## 2. Materials and Methods

Unless otherwise specified, ACS reagent-grade starting materials and solvents were used as received from commercial suppliers without further purification.

Deionized water (resistivity = 18.2 MΩ·cm) obtained from an Aquadem™ system (Veolia Water Technologies, Saint-Maurice, France) was used in all experiments. Hydroxylated nanodiamonds in powder form (DND30, 4 nm average primary particle size, 30 nm average particle size and zeta potential ζ > +30 mV, >98%) were procured from the International Technology Center (Raleigh, NC, USA). Fluorescent hydroxylated nanodiamonds (FND100, >900 NV/particle, 1 mg mL^−1^ H_2_O, 100 nm average particle size, ζ < −20 mV), cetyltrimethylammonium bromide (CTAB, >98%), sodium hydroxide pellets (NaOH, ≥98%), and ammonium nitrate (NH_4_NO_3_, >98%) were purchased from Merck (Saint-Quentin-Fallavier, France). Chlorhydric acid (HCl, ≥37%) was purchased from Thermo Fisher Scientific (Illkirch-Graffenstaden, France). Sodium chloride (NaCl) was obtained from VWR (Radnor, PA, USA). 1,2-Bis(triethoxysilyl)ethylene (ENE, 95%, 80% trans-isomer) was obtained from abcr GmbH (Karlsruhe, Germany). Ammonium hydroxide (28%), absolute ethanol (EtOH), and ethanol (96%) were obtained from Carlo Erba Reagents (Cornaredo, MI, Italy). (6-{2-[2-(2-Methoxy-ethoxy) ethoxy]ethoxy}hexyl)triethoxysilane (PEG-Si, >98%) and 11-aminoundecyltriethoxysilane (AUTES, >97%) were purchased from Sikemia (Montpellier, France). Cell Mask™ Orange Plasma Membrane was obtained from Invitrogen (Cergy-Pontoise, France). Glass-bottom 8-well tissue-culture chambers were purchased from Sarstedt (Marnay, France).

**Analytical techniques**

Samples for transmission electronic microscopy (TEM) measurements were deposited from suspensions on Cu Formvar/C holey grids and left to dry before observation. The main TEM images were acquired using a JEOL 1200 EXII (Tokyo, Japan) operated at 120 kV. The quantification of the nanoparticles’ diameters was performed with ImageJ software with at least 100 counts. The elemental mapping was recorded in STEM mode with an Oxford Instruments XMaxN 100 TLE (100 mm^2^, windowless) EDX detector with a JEOL 2200 FS (FEG) Transmission Electron Microscope operated at 200 kV with a Gatan UltraScan 4000 (4k × 4k) CCD camera (Pleasanton, CA, USA).

Hydrodynamic diameters of the ND@PMO NPs were obtained using a Cordouan Technologies (Pessac, France) DL135 Particle Size Analyzer, and data were treated using NanoQ software using a statistical or a cumulant-based model. Hydrodynamic diameters of the NDs and zeta-potential measurements were recorded on a Malvern NanoSeries (Malvern, UK) Zetasizer NanoZS (model ZEN3600) in a DTS1060C Zetacell (for zeta potentials) at 25 °C, with an equilibration time of 60 s and with automatic measurement, and data were treated using Zetasizer software using a Smoluchowski model. All zeta potentials and hydrodynamic diameters were obtained with nanoparticle suspensions at 100 µg mL^−1^.

Fourier-transform infrared (FTIR) spectra were recorded on a PerkinElmer Spectrum Two (Waltham, MA, USA) spectrometer with four acquisitions at a resolution of 4 cm^−1^.

ND@PMO syntheses were performed at 25 °C using a Fisherbrand™ (Illkirch-Graffenstaden, France) TI-H10 MF2 ultrasonic bath (1000 W, 25 kHz in normal mode).

**Zetaeta potential of NDs**

Briefly, the NDs were suspended in 1 mM NaCl aqueous solutions, yielding a concentration of 100 µg mL^−1^. After quick sonication, pH values were adjusted with 20 or 50 mM NaOH or HCl aqueous solutions, using a pH probe, and the zeta potential (n = 5) was recorded on the Malvern Zetasizer. For each ND suspension, the pH was first fixed at 4, and then it was increased to 8 and 11 for the following measurements.

**Synthesis of ND@ENE PMO core–shell NPs**

Absolute ethanol (5.8 mL), deionized water (10.1 mL), and ammonium hydroxide (NH_4_OH (2 M), 0.6 mL) were added in a 50 mL round bottom flask. Then, an aqueous solution of FND100 (2 mL at 1 mg mL^−1^ and sonicated for 30 min) or DND30 (2 mg, previously dispersed at 1 mg/mL and sonicated for 30 min) was added dropwise under 25 kHz sonication. After 30 min, a surfactant solution of CTAB (80 mg, 0.22 mmol), dissolved in absolute ethanol (0.6 mL) and deionized water (1.3 mL), was added dropwise under powerful sonication. After 60 min of stabilization, 1,2-bis(triethoxysilyl)ethylene (253 µL, 0.72 mmol) was introduced dropwise into the solution under powerful sonication. After 5 min, the resultant suspension was transferred for stirring at 1400 rpm and kept at room temperature overnight.

**Direct grafting of 6-{2-[2-(2-Methoxy-ethoxy) ethoxy]ethoxy}hexyl)triethoxysilane** **(PEG-Si) and 11-aminoundecyltriethoxysilane (AUTES)**

After the 24-h condensation process of core–shell NPs, the temperature was increased to 50 °C. Then, a 2:1 (n:n) mix of PEG-Si (61.6 mg, 150 µmol) and AUTES (25.0 mg, 75 µmol) was dissolved in 1 mL of deionized water and added to the sol–gel preparation. The reaction was subjected to stirring at 1000 rpm and kept at 50 °C overnight. Then, the solution was cooled to room temperature while stirring; fractions were gathered in propylene tubes and collected by centrifugation for 10 min at 14k rpm. Subsequently, the still surfactant-filled particles were solvent-extracted three times with an ammonium nitrate solution (20 g L^−1^ ethanol) in order to remove the CTAB template; then, they were washed two times with ethanol. Each extraction and washing involved 20 min sonication and centrifugation for 10 min at 10k rpm. The nanoparticles were finally redispersed into ethanol and stored at +4 °C.

**Biological studies of NDs and multifunctional ND@ENE NPs**
**Cell line**

A human breast adenocarcinoma (MDA-MB-231 ATCC^®^ CRM-HTB-26™) cell line was purchased from ATCC (Manassas, VA, USA). The cells were cultured in Dulbecco’s Modified Eagle’s Medium (DMEM) supplemented with 10% fetal bovine serum and 50 μg mL^−1^ gentamycin. All cells were allowed to grow in a humidified atmosphere at 37 °C under 5% CO_2_.

**Cell internalization study using confocal microscopy**

MDA-MB-231 cells were seeded into glass-bottom 8-well tissue-culture chambers. A total of 24 h after seeding, cells were treated, or not, with 50 µg mL^−1^ of nanoparticles for 24 h. Fifteen minutes before the end of incubation, cells were treated with CellMask™, Orange Plasma Membrane, Invitrogen (Cergy-Pontoise, France), stain at a final concentration of 5 µg mL^−1^. Cells were washed two times with culture medium before observation with a confocal fluorescence microscope LSM780 (Carl Zeiss, France) at 800 nm for the fluorescent NPs (two-photon excitation) and at 561 nm for the CellMask™ Orange Plasma Membrane stain, using a high magnification (63×/1.4 OIL Plan-Apo).

**Two-photon-excited photodynamic therapy**

MDA-MB-231 cells were seeded into a 384-well glass-bottom plate (thickness = 0.17 mm) with a black polystyrene frame, with 2000 cells per well in 50 μL of culture medium. Then, they were allowed to grow for 24 h. The NPs were dispersed under ultrasonication in PBS at a concentration of 1 mg mL^−1^, and cells were then incubated for 20 h with the NPs at a final concentration of 80 μg mL^−1^ in supplemented DMEM. Nontreated cells were considered as a control. After incubation with the NPs, the cells were maintained in fresh culture medium and then submitted (or not) to TPE with the LSM780 live confocal microscope (Carl Zeiss Microscope). Half of the wells were irradiated at 800 nm by three scans of 1.57 s duration in 4 different areas of the well with a focused laser at maximum laser power (laser power input = 3 W). The laser beam was focused by a microscope objective lens (Carl Zeiss 10×/0.3 EC Plan-Neofluar).

Fifteen minutes before the end of the 2-day incubation, cells were treated with Hoechst 33342™ at a final concentration of 10 µg mL^−1^. Cells were washed two times with culture medium before photo-shooting with a fluorescence microscope LEICA DM IRB at 340/380 nm. The quantification of the living cells was performed with ImageJ software by analyzing pictures of the different wells taken at a magnification of 4×. A specific method was defined and reproduced on all images in an identical way to count the nuclei and to establish the percentage of cell viability according to the following equation:

(number of nuclei in the well/number of nuclei in the control well) × 100,

then, the viability was corrected according to the following formula:

(non-irradiated viability − 2 × (non-irradiated viability − irradiated viability))_._

Data are expressed as mean values with the standard deviation from three independent experiments.

## 3. Results and Discussion

### 3.1. Nanodiamond Characterization

The NDs were characterized before performing in cellulo TPE-PDT to achieve a better understanding of their physicochemical properties.

The hydrodynamic diameter distributions of two different hydroxylated NDs samples were recorded using dynamic light scattering (DLS) measurements after sonication for a couple of minutes. The statistical results showed average sizes in the numbers of 107 and 36 nm, respectively (Table 1). Therefore, the NDs were called FND100 NDs (for the fluorescent NDs) and DND30 NDs (for the detonation NDs).

FND100 NDs are obtained from the phase transformation of graphite at a high pressure and a high temperature (HPHT) by a grinding procedure. Thus, they hold intrinsic fluorescence with at least 900 nitrogen-vacancies (NVs) per particle. DND30 NDs are DNDs synthesized by shocks of trinitrotoluene-like explosives and hexogen prior to the purification and washing steps. TEM images (Figure S2) confirmed the better dispersion of the DNDs’ carbon gems, the synthesis pathway of which is more controlled than that of the HPHT for the FNDs [17].

In addition, the average sizes, calculated from a cumulant-based model, displayed higher hydrodynamic diameters due to the presence of some ND aggregates in the suspensions. In particular, the FND100 NDs presented a high polydispersity index (PDI). Multiple fractionation steps of the FNDs would be required to obtain a narrower size distribution, but this method generally gives very poor yields.

Depending on the synthesis and purification stages, NDs exhibit a large variety of surface functionalities (f.i. -OH, -COOH, -NH_2_, and anhydride). While detonation allows for the introduction of many functional groups, NDs produced via the HPHT pathway predominantly present hydroxyl groups on their surfaces [18]. Thus, in order to obtain better knowledge of the NDs’ surface chemistry, their surface charges were assessed by zeta-potential measurements. The NDs were dispersed in 1 mM NaCl aqueous solution in order to achieve enough conductivity to provide reliable data. At pH 4 and 8, the FND100 NDs displayed a slightly negative surface charge, contrary to the DND30 NDs, which were positively charged (Table 2). The difference between the FNDs’ and the DNDs’ surface charge environment could be associated with the content of different surface functionalities, especially amine and carboxyl moieties that are present in addition to hydroxyl groups. NH_2_ groups are easily protonated into ammonium ions at acidic to neutral pH levels and generally confer very positive zeta-potential values. In comparison, carboxyl groups are responsible for a negative surface charge upon deprotonation (–COO^−^), even at a low pH [19]. According to this behavior, $\frac{R-\mathrm{COOH}}{R-\mathrm{NH}2}$ and $\frac{R-\mathrm{COOH}}{R-\mathrm{OH}}$ ratios could be higher at an FND’s surface than at a DND’s surface. This is confirmed at pH ≈ 11, where the NDs are all negatively charged, with an absence of ammonium and a predominance of OH and COO^−^. Similar results were obtained by Khanal and coworkers for hydroxylated DNDs. Indeed, the positive zeta potential of the NDs was correlated with N_1s_ X-ray photoelectron spectroscopy measurements, which revealed the presence of a high nitrogen content [20,21].

### 3.2. Syntheses of ND@PMO Nanoparticles and Associated Grafting Strategy

FND100 NDs and DND30 NDs were encapsulated in biocompatible PMO shells via a specific sol–gel process to enhance their biocompatibility while maintaining their potential for ROS generation under TPE-PDT conditions.

The first challenge of this strategy was to deal with the spontaneous aggregation of carbon-based materials in aqueous media, which is mainly due to van der Waals forces [22]. The reaction mixture was indeed basic as ammonium hydroxide was used as the sol–gel catalyst and CTAB cationic surfactant was applied as a porogen agent. To address this, we adapted a reported procedure for the preparation of core–shell nanodiamond–mesoporous silica nanoparticles (ND@MSNs) where the continuous sonication of the mixture reaction allows for the better dispersion of NDs in CTAB micelles in order to limit particle-size polydispersity issues [23,24]. ND@PMO core–shell nanocapsules were then prepared via a modified Stöber process. The addition of organoalkoxysilane precursors under ultrasonication in a water bath (1000 W, 25 kHz) leads to shell growth around NDs by concomitant condensation and hydrolysis. Notably, without any sonication during the precursor addition, very monodisperse PMO NPs and very large ND aggregates (covered, or not, with a small silica layer) were visible by transmission electronic microscopy (TEM) imaging.

**Figure 1:**
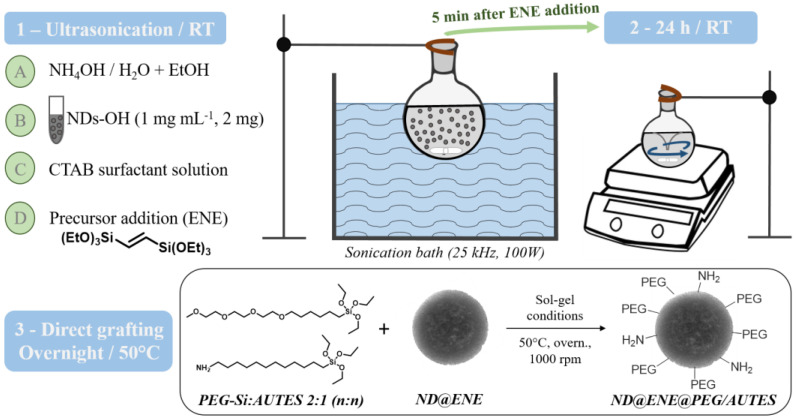
Synthesis and functionalization steps of ND@ENE NPs.

Different ND@PMO NPs were synthesized and functionalized using a one-pot strategy. Core–shell nanosized particles were formed under ultrasonication for 5 min. The reaction mixture was removed from the bath, and the NPs were allowed to grow for 24 h under stirring (Figure 1, Steps 1–2). Then, direct grafting of short-chain silylated polyethylene glycol (PEG-Si, M_w_ = 410.62 g mol^−1^) and aminoundecyltriethoxysilane (AUTES, M_w_ = 333.59 g mol^−1^) was performed using an ENE:PEG-Si:AUTES molar ratio of 9.6:2:1 (Figure 1, Step 3).

**Figure 2:**
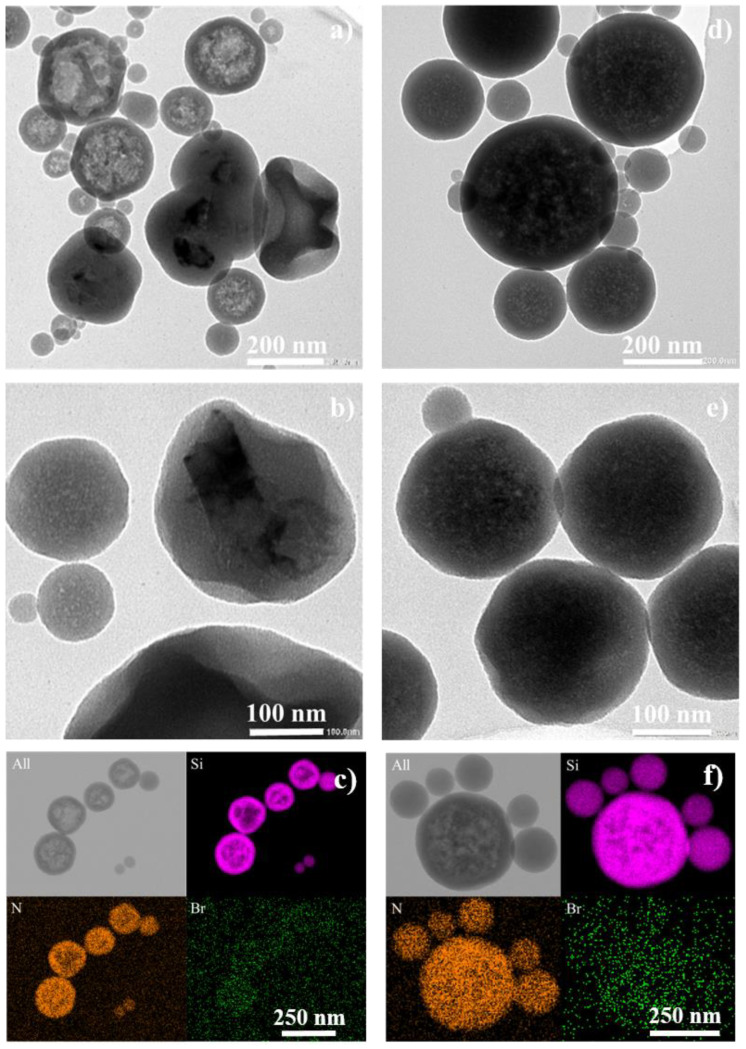
TEM and STEM-EDX micrographs of ND@ENE@PEG/AUTES NPs: (**a**,**b**) Spherical nanocapsules containing multiple FND100 NDs or distorted core–shell NPs with large FND100 NDs; (**c**) The corresponding STEM-EDX elemental mapping; (**d**,**e**) Spherical nanocapsules containing multiple DND30 NDs; and (**f**) The corresponding STEM-EDX elemental mapping. PMO NPs smaller than 60 nm do not possess NDs.

ND@ENE@PEG/AUTES hydrodynamic sizes were investigated by DLS measurements, by using either Padé-Laplace (statistical size) or cumulant-based algorithms. FND100 ND- and DND30 ND-based NPs revealed low PDIs and similar diameters, with z-average values of 339 and 323 nm, respectively (Table 3). TEM imaging showed more polydisperse objects with diameters of 137 ± 83 and 147 ± 86 nm for FND100@ENE- and DND30@ENE-based samples, respectively (Figure 2a–d).

To go further, TEM micrographs revealed that FND100@ENE NPs display either spherical nanocapsule-like or distorted spherical core–shell morphologies with large FNDs with flake shapes (Figure 2a,b). Variable aggregate sizes are responsible for the polydisperse sizes of the final ND@PMO nanocapsules. Moreover, by screening the different sizes of NP populations, it was observed that NPs with a diameter below 60 nm were not encapsulating NDs (Figure 2a,d), even in the case of small DND30 NDs; thus, these objects were simple PMO NP spheres. This could be explained by the heterogeneous repartition of NDs in micelles before initiating the sol–gel process and by the low proportion of NDs used in the preparation mixture in order to avoid aggregation issues, resulting in the condensation of ENE precursors without any ND core in the micelle. Note that, while DLS investigation demonstrates that the systems are homogeneous at the macroscopic scale, TEM only shows a sample of nanoparticles and serves to elucidate the different textures of nanoparticles.

We then investigated the functionalization of the ND@PMO NPs with PEG-Si and AUTES via several characterization techniques.

Zeta-potential measurements using the DLS principle allowed for the validation of PEG-Si and AUTES grafting. It is well-known that pristine silica NPs exhibit strongly negative surface charges, because of the large proportion of silanolate (Si-O^−^) on their external surfaces. Due to the protonation of amine moieties to generate NH_3_^+^ ions, functionalized FND100@ENE and DND30 NPs showed very positive surface charges, with zeta-potential values of +19.7 and +26.0 mV, respectively.

Scanning transmission electron microscopy energy-dispersive X-ray spectroscopy (STEM-EDX) elemental mapping of ND@ENE@PEG/AUTES NPs (Figure 2c–f) revealed the presence of nitrogen on the PMO surface, which suggests AUTES grafting. Additionally, the presence of a low amount of bromide confirms that CTAB was properly removed (after synthesis and surface modification) by the NH_4_NO_3_ extraction and washing steps using an ion exchange technique between the nitrate NO_3_^−^ and Br^−^ ions.

Finally, bands corresponding to the covalent grafting of PEG-Si and AUTES were identified via infrared spectroscopy (Figure S3). For pristine ND@ENE NPs, the signals at 2977, 1634, and 922 cm^−1^ correspond to the C=C bond in the ENE bridges. Furthermore, the appearance of asymmetric and symmetric stretching vibration modes of aliphatic carbons (ν_CH2_) at 2929 and 2859 cm^−1^ confirmed the coupling of both PEG-Si and AUTES. At 1350 cm^−1^, another symmetrical stretching mode of alkane (δ_CH2_) was detected.

### 3.3. Biological Studies of Functionalized ND@ENE Nanoparticles

#### 3.3.1. TPEF Imaging of NDs and ND@ENE

**Figure 3:**
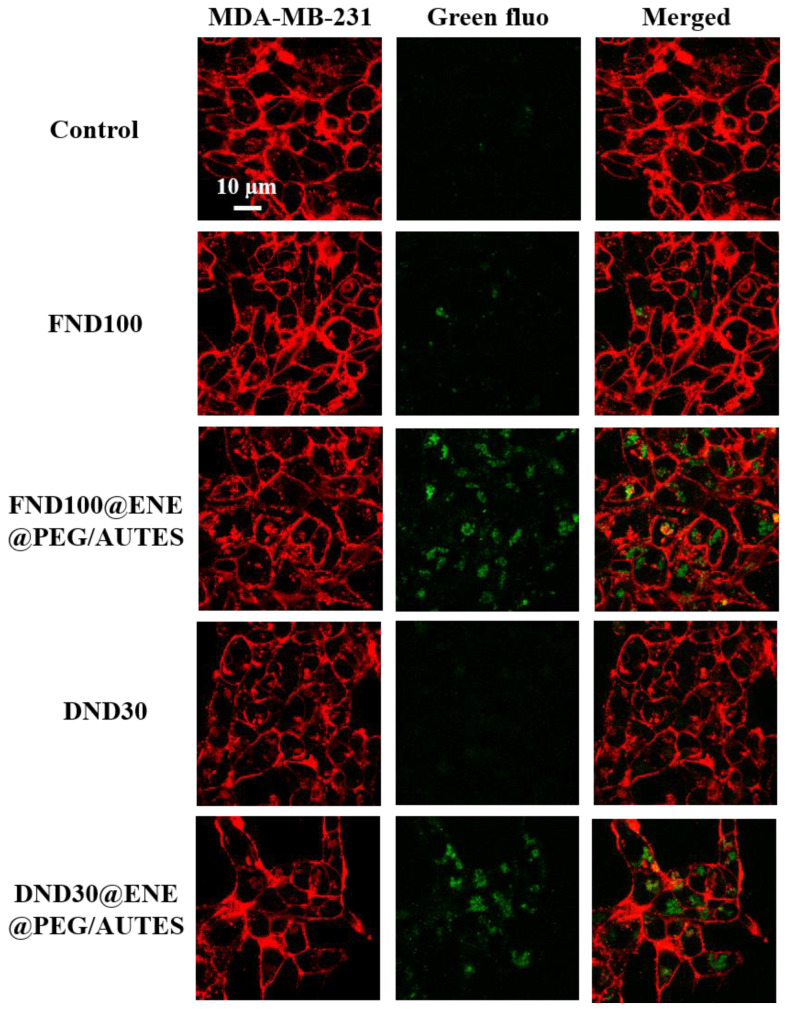
Cell internalization study of different functionalized ND@ENE core–shell NPs and NDs at 50 µg mL^−1^ using confocal microscopy imaging. MDA-MB-231 cancer cells were stained with Cell Mask™ Orange Plasma Membrane stain after incubation for 20 h and then visualized at 561 nm (membrane) or 800 nm (NPs, green fluorescence). Images were obtained with a Carl Zeiss LSM780 confocal fluorescence microscope using a high magnification (63×/1.4 OIL Plan-Apo).

The internalization of NPs into cancer cells was screened by two-photon-excited fluorescence (TPEF) imaging. NPs were incubated at 50 µg mL^−1^ for 20 h. Fifteen minutes before the imaging experiments, the cell membranes were stained with Cell Mask™ Orange Plasma Membrane stain. All NPs were successfully endocytosed by the cancer cells, as shown by the TPEF appearance at 800 nm inside the cytosol (Figure 3). The remarkable level of internalization observed for the FND100@ENE@PEG/AUTES and the DND30@ENE@PEG/AUTES NPs compared to NDs can be associated with the suitable interactions between the negatively charged MDA-MB-231 cells and the positively charged NPs due to surface amine functionalities.

#### 3.3.2. Phototoxicity Studies

**Figure 4:**
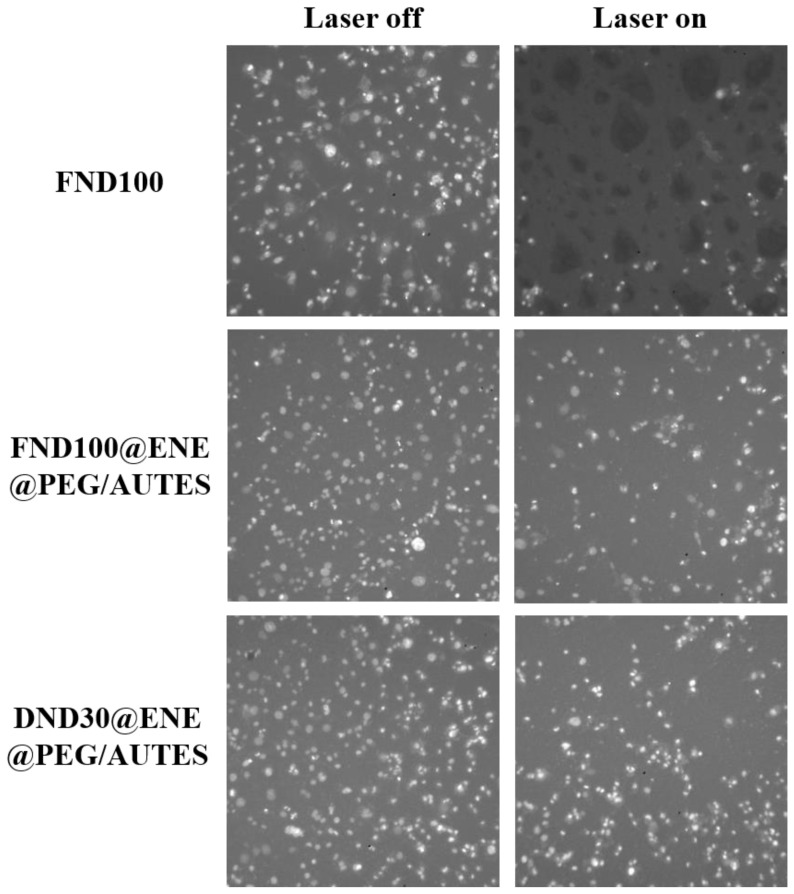
Images of wells obtained by fluorescence microscopy. Extracted images represent well areas that have been subjected (or not) to two-photon irradiation (3 × 1.57 s) at 800 nm. White dots correspond to living MDA-MB-231 cancer cells.

After treating the cells with the NPs, the cells were irradiated (3 × 1.57 s), or not, at 800 nm after 24 h of incubation. Then, cell death was assessed 48 h after irradiation by nuclear labelling with Hoechst 33342™, which is a DNA intercalant that stains the nuclei of living cells with a blue color. Thus, the phototoxicity of the ND@PMO@PEG/AUTES and corresponding nanodiamond NPs towards MDA-MB-231 breast cancer cells was studied. For all samples, cancer cells were incubated with NPs at 80 µg mL^−1^ for 20 h. Figure 4 illustrates some images of the wells after staining and from which the data were collected after the counting of the living cells.

**Figure 5:**
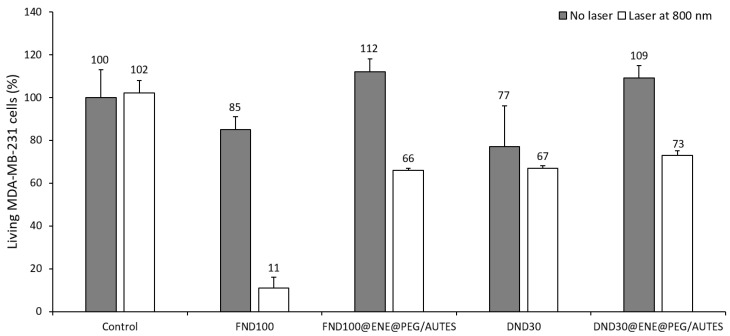
TPE-PDT studies of ND@ENE@PEG/AUTES NPs and the corresponding NDs. TPE-triggered cancer-cell killing (MDA-MB-231 epithelial human breast) after incubation with NPs at 80 µg mL^−1^ for 20 h and irradiation at 800 nm (3 × 1.57 s). Lines represent the standard deviations from three independent experiments.

As revealed in the well image after irradiation, bare FND100 were found to be very efficient, causing 74% of cell death. Interestingly, when NDs were encapsulated at a low concentration level in an ENE shell covered by PEG-Si and AUTES, the TPE-PDT killing efficiency was remarkable, with 46 and 36% mortality, respectively, with FND100- and DND30-based nanoparticles (Figure 4 and Figure 5), in agreement with the excellent internalization of these core–shell systems. While NDs alone presented cytotoxicity with no irradiation, encapsulated NDs revealed high biocompatibility, which can be associated with the protective organosilica matrix. Altogether, these results suggest that NDs with different morphologies and sizes are suitable for TPE-PDT, but also that a protective shell is necessary to enhance biocompatibility and ensure endocytosis through surface functionalization.

## 4. Conclusions

Fluorescent or detonation-hydroxylated nanodiamonds of different sizes and morphologies were encapsulated in a biocompatible PMO shell using 1,2-bis(triethoxysilyl)ethylene as precursor of the sol–gel process under ultrasonication for a better disaggregation of the ND gems in order to obtain better core–shell yields and a more monodisperse colloidal suspension. Two-photon confocal imaging of the prepared ND@ENE core–shell systems functionalized with short PEG and amine functions revealed efficient internalization in MDA-MB-231 cancer cells. Furthermore, biological testing upon TPE-PDT showed their high biocompatibility and noteworthy levels of cell-killing ability after irradiation, with respectively 46% and 36% cell death for FND100- and DND30-based core–shell nanoparticles.

The results presented in this work pave the way for the further use of ND–organosilica core–shell-based systems for TPE-PDT. The exploration of the ND structure–performance relationship, especially regarding the surface environment, size, and morphology, could be achieved to obtain optimal photophysical properties. Furthermore, different processes could be tested to build multifunctional ND@PMO structures in order to determine the configuration in which the photosensitizing effect could be the most powerful, while monitoring ND intrinsic biocompatibility without irradiation. Altogether, with large PMO-shell porosities, such multifunctional ND@PMO materials could be suitable nanoplatforms for targeted combinatory TPE-PDT/chemotherapy with selective drug delivery.

## Figures and Tables

**Table 1:** Size measurements of commercial hydroxylated NDs: hydrodynamic diameters (in nm) in number or intensity. Measurements were performed at 100 µg mL^−1^ in water.

Samples	Statistical Size (Number)	Average Size (Intensity)	PDI
FND100	107 ± 32	231	0.27
DND30	36 ± 16	88	0.18

**Table 2:** Zeta-potential measurements of commercial hydroxylated NDs (in mV). Zeta potential (1 mM NaCl aqueous solutions) with pH adjusted by adding aliquots of HCl or NaOH.

Samples	pH 4	pH 8	pH 11
FND100	−11.3 ± 0.3	−12.2 ± 0.2	−15.6 ± 0.7
DND30	+36.6 ± 0.6	+33.4 ± 1.0	−5.4 ± 0.3

**Table 3:** Size measurements of different ND@ENE@PEG/AUTES NPs (in nm). ^a^ Statistical hydrodynamic diameter. ^b^ z-average hydrodynamic diameter, n = 3 independent experiments. PDI ≤ 0.20 indicates good-size monodispersity. ^c^ Average diameters of n ≥ 100 counts determined on TEM images. Hydrodynamic diameters were determined from suspensions in ethanol.

Core–Shell NPs	Statistical Size ^a^ (Intensity)	Average Size ^b^ (Intensity)	PDI	TEM ^c^
FND100@ENE@PEG/AUTES	350 ± 40	339	0.11	137 ± 83
DND30@ENE@PEG/AUTES	342 ± 50	323	0.14	147 ± 86

## Data Availability

Not applicable.

## Supplementary Materials

The following supporting information can be downloaded at: https://www.mdpi.com/article/10.3390/life12122044/s1, Figure S1: Energy-level diagram of nitrogen vacancy (NV^−^) centers contained in NDs; Figure S2: TEM micrographs of hydroxylated NDs; Figure S3: Infrared spectra of FND100@ENE and FND100@ENE@PEG/AUTES PMO NPs.
