# Supplementary material for: Photosensitivity of Different Nanodiamond–PMO Nanoparticles in Two-Photon-Excited Photodynamic Therapy
Source: Life (Basel). 2022 Dec 7;12(12):2044. doi: 10.3390/life12122044 (PMC9781408; doi:10.3390/life12122044)
Supplement: Supplementary file 1 [file life-12-02044-s001.zip › life-2072240-supplementary.pdf]

Supplementary Information

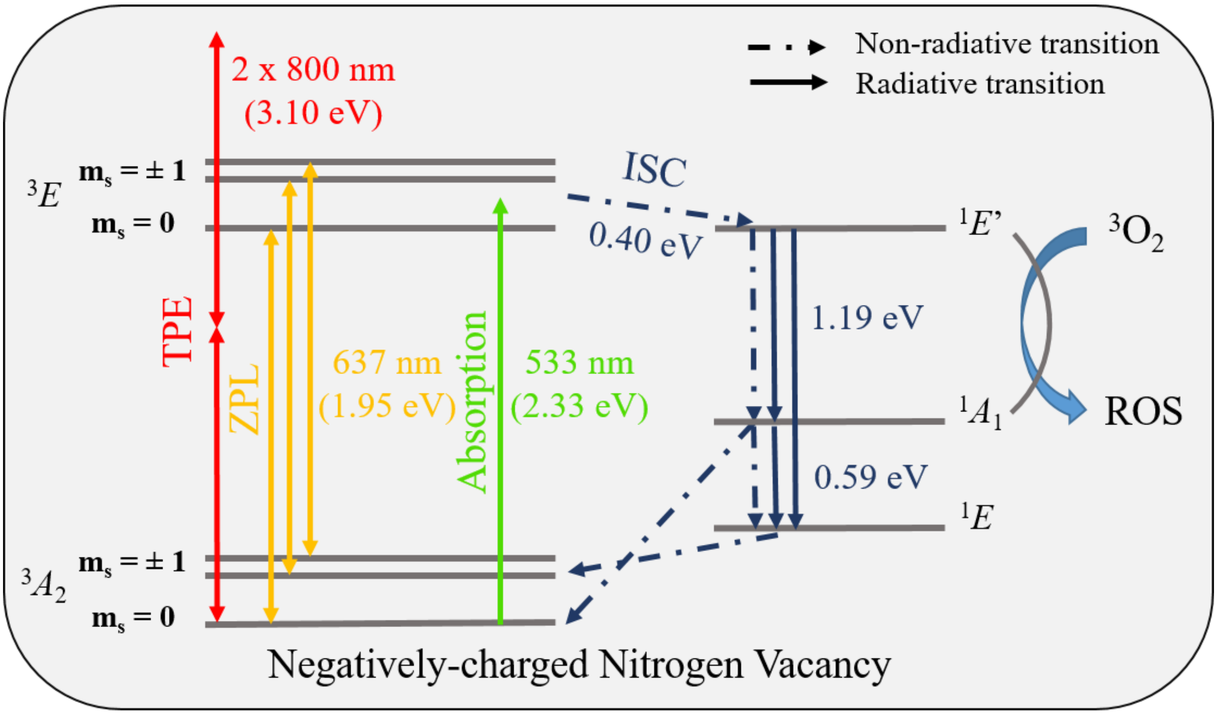

Figure S1. Energy-level diagram of negative nitrogen vacancy (NV^-) centers contained in nanodiamonds. ISC: Intersystem Crossing; ROS: Reactive oxygen species; TPE: Two-photon excitation; ZPL: Zero-phonon line. Adapted from reference [1].

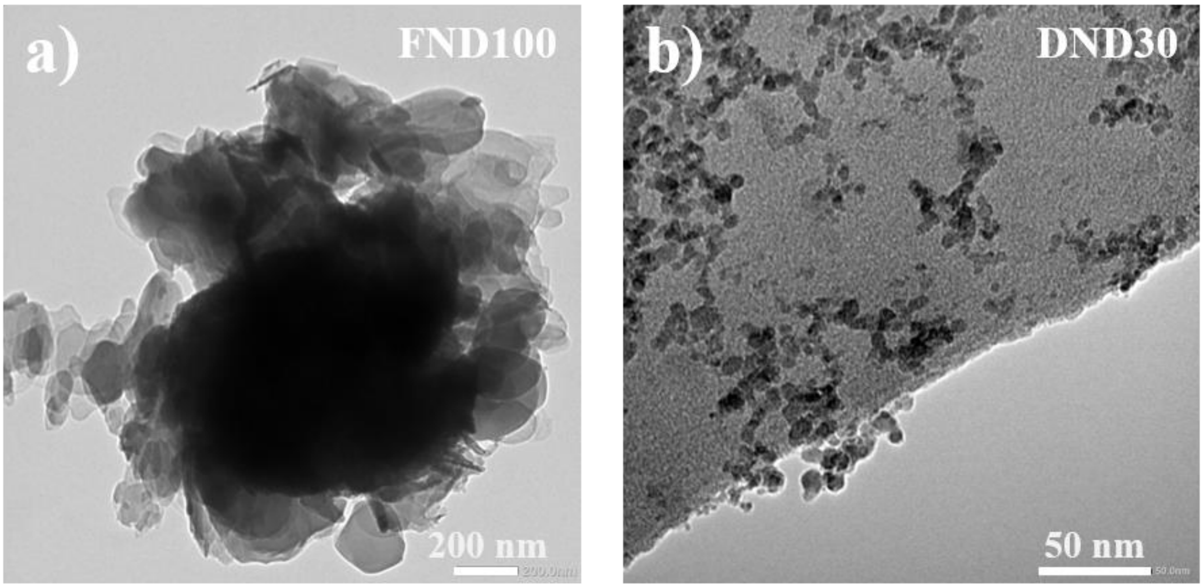

Figure S2. TEM micrographs of hydroxylated NDs. (**a**) FND100; (**b**) DND30.

Supplementary Information

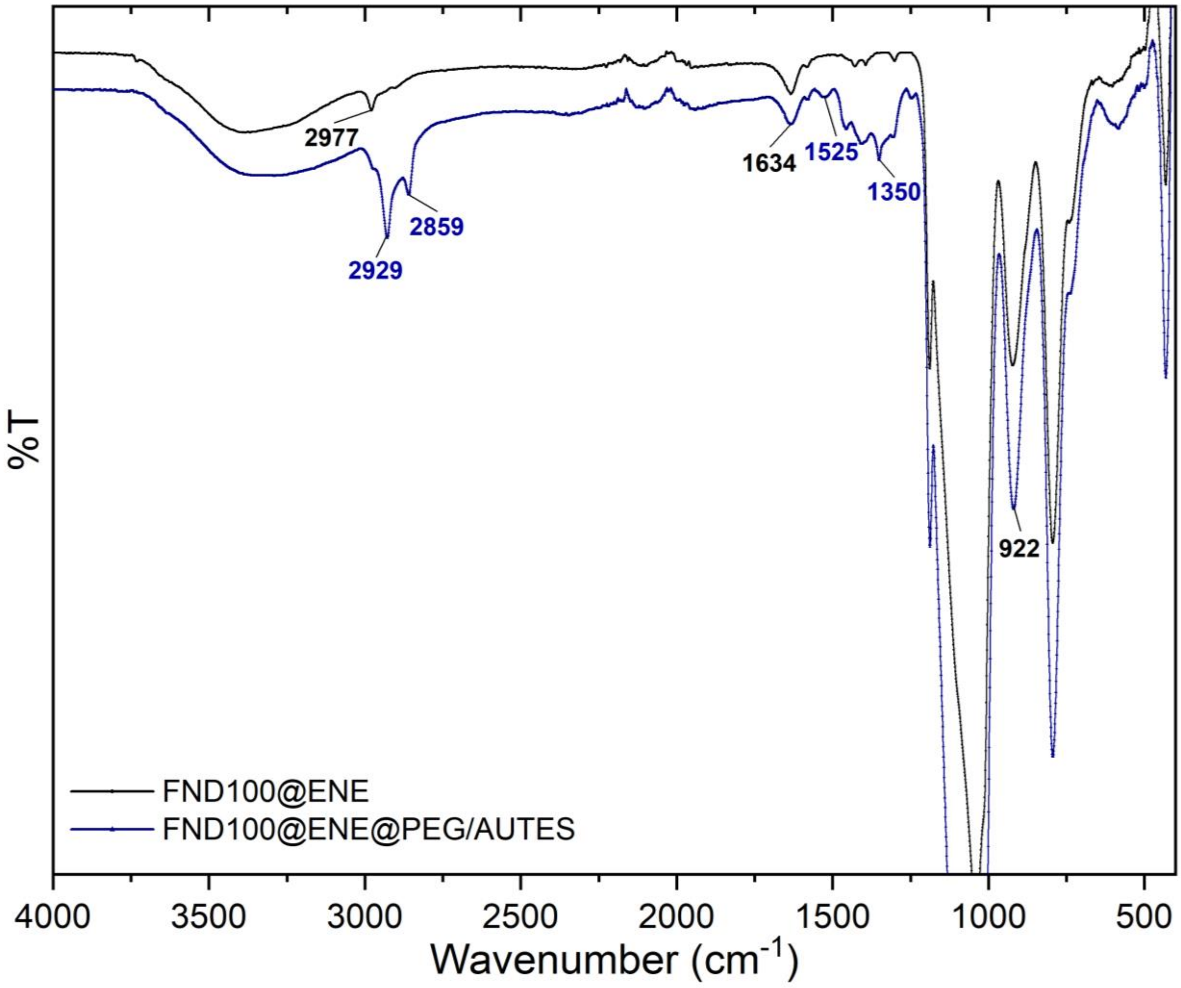

Figure S3. Infrared spectra of FND100@ENE and FND100@ENE@PEG/AUTES PMO NPs.

References

1. Ma, Y.; Rohlfing, M.; Gali, A. Excited states of the negatively charged nitrogen-vacancy color center in diamond. *Physical Review B* **2010**, *81*, 041204, doi:10.1103/PhysRevB.81.041204.
